# Urgent Cesarean Section in a Patient on Venovenous Extracorporeal Membrane Oxygenation

**DOI:** 10.7759/cureus.52046

**Published:** 2024-01-10

**Authors:** Daniela Havens-Lastarria, Sara K Biladeau, Daniel Haines, Ryan Grell

**Affiliations:** 1 Anesthesiology and Perioperative Medicine, University of Louisville School of Medicine, Louisville, USA

**Keywords:** complications and outcomes on gravid and post-partum patients on ecmo, delivery during ecmo support, placental abruption, acute hypoxemic respiratory failure, cesarian section, vv ecmo

## Abstract

A G7P6 40-year-old female at 20 weeks gestation, with a history of polysubstance use disorder and hepatitis C, presented to the emergency department with severe shortness of breath and hypoxia requiring intubation. After a thorough workup, she was diagnosed with aspiration pneumonitis and was treated with a course of antibiotics. After progressing well, she was soon extubated and transferred to a subacute rehabilitation facility (SAR). There, she acutely decompensated, requiring readmission, reintubation, and venovenous extracorporeal membrane oxygenation (ECMO) cannulation. After a brief period of improvement, the patient became increasingly unstable with hypotension, anemia, and downtrending fibrinogen. Bedside imaging indicated a possible placental abruption. After extensive discussion among the care teams and patient's healthcare proxy, an urgent cesarean section was performed. Although the fetus was determined to be nonviable, the patient tolerated the procedure well and was eventually decannulated from ECMO and transferred to a SAR.

## Introduction

Since it was first introduced in 1970, the indications for ECMO have gradually expanded from cardiopulmonary bypass support in neonates to applications in the adult population. Although around 12,000 adults are now placed on ECMO each year, fewer than 150 of these patients are in the peripartum period [1]. While mainly used as a salvage therapy of last resort in pregnancy, ECMO does confer risks to the viability of the fetus. This is a case report of a 40-year-old female who at 20 weeks gestation was placed on ECMO for hypoxic respiratory failure then suffered a placental abruption and was taken to the operating room for an urgent cesarean section.

This article’s abstract was previously presented as a poster at the Society of Cardiovascular Anesthesiologists Annual Meeting in Portland, Oregon, in May 2023.

## Case presentation

**Figure 1:**
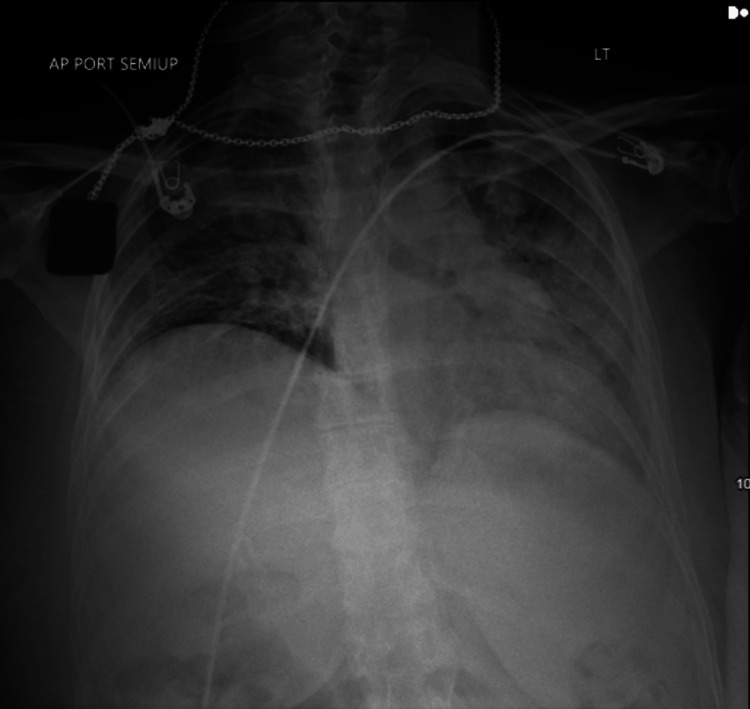
Anterior-Posterior Semi-Upright Chest X-ray Obtained Upon Admission

The patient was a G7P6 40-year-old female at 20 weeks gestation with a history of polysubstance use disorder and hepatitis C who presented to the emergency department with severe shortness of breath and hypoxia. She described experiencing progressive shortness of breath over the past few days after a probable heroin overdose. Upon physical examination, she was noted to be 77 kg in weight and 167 cm in height and appeared to be in severe distress. Her cardiac examination was unremarkable; however, there were severe bilateral crackles present in all lung fields. Prior to the application of supplemental oxygen, her oxygen saturation was noted to range between 70-75%. She was quickly placed on a non-rebreather mask connected to 100% fraction of inspired oxygen (FiO2) at a flow rate of 15 L per minute with a marginal improvement of her oxygen saturation to a SpO2 of 80-85%. An arterial blood gas was collected and was notable for a hemoglobin of 10.1 g/dL, a pH of 7.4, pCO2 of 45 mmol/L, and a PaO2 of 56 mm of 100% FiO2. A portal chest X-ray (Figure 1) was obtained which revealed bilateral interstitial and airspace opacities that were consistent with noncardiac pulmonary edema versus diffuse pneumonia.

During her emergency department visit, she became progressively tachypneic and was intubated for impending respiratory failure when her respiratory rate reached 30 breaths per minute. After intubation, a bronchial alveolar lavage (BAL) was ordered, and the patient was started on the broad-spectrum antibiotics ceftriaxone and vancomycin. She was then transferred to the intensive care unit (ICU) for further observation and management.

Over the next five days, a significant workup by the pulmonology, infectious disease, rheumatology, and obstetric services was completed and was notable only for protein S deficiency, MTHFR mutation, and a positive initial BAL for pulmonary macrophages and occasional neutrophils with no bacterial growth after five days. This led to a working diagnosis of aspiration pneumonitis.

After approximately five days of antibiotic and supportive treatment, she was extubated and transferred to an intermediate care unit in stable condition. Two days later, she was transferred to the medical ward, her antibiotics were stopped, and she was discharged to a nearby subacute rehabilitation facility.

Within hours of presenting at the rehabilitation facility, however, she acutely decompensated and was transferred back to the ICU, where she was emergently intubated. Despite escalating ventilator management, the patient became increasingly hypercapnic and acidotic with a pH of 6.9 and a PaCO2 of > 105 mmHg. A transthoracic echocardiogram (TTE) was performed, which indicated normal biventricular function without hemodynamically significant valvulopathies. Subsequently, the decision was made to proceed with venovenous (VV) extracorporeal membrane oxygenation (ECMO) cannulation via a femoral-femoral approach.

After an uneventful cannulation, the patient stabilized and demonstrated clinical and laboratorical improvement for approximately three days. On Day 4 of VV ECMO, the patient became increasingly unstable, manifesting hypotension, anemia, and downtrending fibrinogen. Bedside imaging indicated a finding of placental lakes, which raised concerns of placental abruption. After extensive discussion among the care teams and the patient's healthcare proxy, an urgent cesarean section was planned in order to provide the patient with the best chance at survival.

An anesthesia team consisting of two anesthesiologists - one obstetric and one cardiothoracic - and a fourth-year anesthesia resident physician was selected to care for the patient intraoperatively. After bringing the patient to the operating room and connecting her to the American Society of Anesthesiologists’ standard monitors and an anesthesia machine, two large-bore IVs and a radial arterial line were placed. After the catheters were confirmed to be functioning correctly, the patient’s propofol infusion was up-titrated from her existing ICU sedation dose of 50 mcg/kg/min to 100 mcg/kg/min and an additional bolus of 50 mg was administered. Then, the surgeons prepped the patient’s abdomen in the usual fashion and quickly delivered the fetus via a low transverse incision.

The fetus was transferred to a waiting team of neonatologists and was determined to be nonviable. The placenta was removed from the uterus and hemostasis was achieved with the aid of 26 units of IV oxytocin (two boluses of three units spaced three minutes apart, with 20 units infused over one hour) and 250 mcg IM carboprost. One liter of blood loss was noted; however, the patient remained hemodynamically stable throughout the operation and was returned to the ICU postoperatively for close monitoring.

The remainder of her course included recanalization of ECMO from bilateral femoral cannulation to a dual-lumen right internal jugular catheter in order to facilitate physical conditioning, a tracheostomy due to failure to wean from the ventilator, eventual decannulation of the ECMO circuit, and discharge to the same subacute rehabilitation.

## Discussion

The uterus is a vascular organ, receiving almost 1 liter of blood per minute at term, which conveys the potential for significant blood loss during delivery [2]. According to the American College of Obstetrics and Gynecologists, significant blood loss becomes classified as a postpartum hemorrhage (PPH) in both vaginal deliveries and cesarean sections when blood loss totals one liter or when any amount of blood loss is accompanied by signs and symptoms of hypovolemia [3]. Placental abruption, a common cause of PPH, occurs when the placenta prematurely separates from the uterus at the decidual-placental interface, thus shearing maternal uterine vessels and leading to blood accumulation between the placenta and uterus [2,4]. Typically, the volume of blood loss caused by the abruption is limited by self-tamponade; however, the separation of the maternal blood supply from the fetus can deprive the fetus of oxygen and other nutrients, leading to fetal demise. If the condition goes unrecognized and spontaneous delivery is allowed to progress, the abruption can quickly progress to a PPH once the self-tamponade is relieved. Additionally, as with any significant bleeding, the coagulation cascade is activated and coagulation factors are quickly consumed, which can ultimately lead to disseminated intravascular coagulopathy (DIC), another life-threatening concern for the parturient. Risk factors for placental abruption tend to be associated with a decrease in uterine blood flow due to vasoconstriction and include prior abruptions, smoking, trauma, cocaine use, multifetal gestation, preterm premature rupture of membranes, intrauterine infections, hypertension, preeclampsia, thrombophilia, advanced maternal age of greater than 35 years old, and hydramnios [2,4].

The mechanism for extracorporeal membrane oxygenation (ECMO) was derived from the original heart/lung machine in 1953, although the original setup was incapable of providing prolonged support [5]. Various improvements lead to better outcomes and subsequent expansion of the use of ECMO to a broader patient population (Tables 1-2). To be considered for ECMO, a patient must demonstrate inadequate pulmonary function with regard to oxygenation and other gas exchange; this may or may not be accompanied by inadequate cardiac output. Accordingly, there are two modern types of ECMO: venovenous (VV), which supports the lungs only, and venoarterial (VA), which supports both the heart and lungs.

**Table 1:** Indications for VV ECMO VV: Venovenus; ECMO: extracorporeal membrane oxygenation; ARDS: acute respiratory distress syndrome; TRALI: transfusion-related acute lung injury; PE: pulmonary embolism

Common Indications for VV ECMO	Expanded Indications for VV ECMO
ARDS with preserved cardiac function	Penetrating chest trauma with TRALI
Severe air leak syndromes	Posttraumatic ARDS
Diffuse alveolar hemorrhage	Mediastinal masses
Severe asthma	Perioperative setting for pulmonary thromboendarterectomy
Severe pulmonary contusions	Airway surgery in patients with high risk of airway collapse
Severe inhalational injury	Rewarming patients with profound accidental hypothermia
Peri-lung transplant respiratory failure	Acute pH crises to indirectly support the right heart (PE, endarterectomy, left-to-right intracardiac shunting)

**Table 2:** Indications for VA ECMO VA: venoarterial; ECMO: extracorporeal membrane oxygenation; ARDS: acute respiratory distress syndrome; eCPR: extracorporeal cardiopulmonary resuscitation

Common Indications for VA ECMO	Expanded Indications for VA ECMO
Myocardial infarction	Acute cardiogenic shock in the setting of chronic heart failure
Myocarditis	Refractory ventricular arrhythmias
Post-cardiotomy heart failure (failure to wean from cardiopulmonary bypass)	eCPR
ARDS with severe cardiac dysfunction	Primary graft dysfunction after heart transplantation
Cardiac trauma	Pulmonary embolism
Acute anaphylaxis	Periprocedural support

The basic ECMO circuit consists of inflow and outflow cannulas (named for the flow directionality in relation to the ECMO circuit), tubing, a pump, and a membrane oxygenator/heat exchanger. ECMO systems use venous inflow cannulas that transport deoxygenated blood from the patient to the oxygenator and outflow cannulas that return oxygenated blood to either the venous system (VV ECMO) or arterial system (VA ECMO) [5]. Regardless of the type of support provided, ECMO is meant to be a temporary support measure as a bridge to one of three outcomes: recovery, a permanent solution such as a ventricular assist device (VAD) or transplant, or withdrawal of care.

In order to prevent circuit thrombosis and to prolong the lifespan of the oxygenator, anticoagulation therapy must be administered to the patient. Unfractionated heparin is the most commonly utilized agent used for this purpose due to its rapid onset, ready availability, prescriber comfort, and known antagonists [5]. As with most therapies that require anticoagulated patients, bleeding is the most common side effect of ECMO, occurring in 10-30% of patients utilizing the therapy [6]. Although most often observed at the cannulation sites, bleeding is also commonly seen in the airway, bladder, and gastrointestinal mucosa. Because of this bleeding, anemia, hemolysis, thrombocytopenia, and heparin-induced thrombocytopenia (HIT) are also relatively common complications in patients connected to ECMO. Other common complications include renal failure, bacterial pneumonia, sepsis, and neurological complications such as seizures and cerebral hemorrhage.

As expected, patients on ECMO are at high risk for bleeding with invasive procedures. In addition to heightened risk due to patient anticoagulation, patients on ECMO often suffer from platelet dysfunction and thrombocytopenia [5]; however, the risks of a significant hemorrhage are relatively low, even among parturients, although few cases of mild to moderate vaginal bleeding and rare instances of catastrophic PPH have been reported among parturients on ECMO [7-8]. The risk of bleeding, however, should not deter the initiation of ECMO in patients who meet the initiation criteria, as the potential benefits of improving oxygenation are almost always more important in determining patient survivability. Indeed, some sources even recommend the routine initiation of ECMO soon after mechanical ventilation is established in parturients [1].

Perioperative assessment of any patient on ECMO necessitating an invasive surgical procedure should include a thorough history, physical, and assessment of any frequent complications specific to ECMO support, including anemia, bleeding, venous thrombosis, and DIC or other coagulopathies. Careful attention should be given to the anesthetic plan, as well as current ECMO settings and inflow pressures [9]. Prior to the transport of patients to the operating room, the ECMO circuit should be carefully inspected to assess for fibrin depositions or thrombus; if a heavy burden is found, there should be a discussion between the ICU and surgical teams to consider an oxygenator exchange. Perioperative transthoracic or transesophageal echocardiography should be considered to assess for cardiac dysfunction, proper ECMO cannula placement, and potential thrombi. Blood products should be available in the OR prior to the start of the procedure.

Total intravenous anesthesia (TIVA) is the preferred method of anesthetic delivery in the general ECMO population because bypassing the pulmonary circulation either partially (as in VV ECMO) or completely (in VA ECMO) makes the uptake of volatile anesthetic agents unpredictable. In patients undergoing a cesarean section while on VV ECMO, however, inhalational agents may be used to facilitate uterine relaxation if needed. In these cases, timely conversion to TIVA should be considered post-delivery to facilitate adequate uterine tone. For patients who arrive at the OR already sedated, much less anesthetic may be required; therefore, close monitoring and titration are necessary [10-12].

## Conclusions

ECMO has been shown to be a viable salvage therapy for reversible forms of cardiorespiratory failure, including for severe acute respiratory distress syndrome when all other therapeutic strategies have failed, and should not be withheld during the peripartum period. In fact, some sources even recommend the routine initiation of ECMO soon after mechanical ventilation is established in these patients. The risk of gynecological bleeding is considered to be limited, although a few cases of mild-to-moderate vaginal bleeding and rare instances of catastrophic PPH have been reported. In this patient, concurrent VV ECMO, placental abruption, and concerns for DIC further complicated decision-making and increased the likelihood of PPH. Overall, however, in thoughtfully selected patients, ECMO is considered to present low risks to maternal and fetal wellbeing.

## References

[1] Webster CM, Smith KA, Manuck TA (2020). Extracorporeal membrane oxygenation in pregnant and postpartum women: a ten-year case series. Am J Obstet Gynecol MFM.

[2] Mayer DC, Spielman FJ, Bell EA (2019). Antepartum and postpartum hemorrhage. Chestnut's Obstetric Anesthesia: Principles and Practice, 6th ed.

[3] Committee on Practice Bulletins-Obstetrics (2017). Practice Bulletin No. 183: postpartum hemorrhage. Obstet Gynecol.

[4] Oyelese Y, Ananth CV (2006). Placental abruption. Obstet Gynecol.

[5] Gutsche JT, Ramakrishna H, Seelhammer TG (2023). Extracorporeal membrane oxygenation. Kaplan’s Cardiac Anesthesia: Perioperative and Critical Care.

[6] Lorusso R, Barili F, Mauro MD (2016). In-hospital neurological complications in adult patients undergoing venoarterial extracorporeal membrane oxygenation: results from the Extracorporeal Life Support Organization Registry. Crit Care Med.

[7] Anselmi A, Ruggieri VG, Letheulle J (2015). Extracorporeal membrane oxygenation in pregnancy. J Card Surg.

[8] Sharma NS, Wille KM, Bellot SC, Diaz-Guzman E (2015). Modern use of extracorporeal life support in pregnancy and postpartum. ASAIO J.

[9] Fierro MA, Daneshmand MA, Bartz RR (2018). Perioperative management of the adult patient on venovenous extracorporeal membrane oxygenation requiring noncardiac surgery. Anesthesiology.

[10] Naoum EE, Chalupka A, Haft J (2020). Extracorporeal life support in pregnancy: a systematic review. J Am Heart Assoc.

[11] Pacheco LD, Saade GR, Hankins GD (2018). Extracorporeal membrane oxygenation (ECMO) during pregnancy and postpartum. Semin Perinatol.

[12] Agerstrand C, Abrams D, Biscotti M (2016). Extracorporeal membrane oxygenation for cardiopulmonary failure during pregnancy and postpartum. Ann Thorac Surg.

